# Continuity of Visual and Auditory Rhythms Influences Sensorimotor Coordination

**DOI:** 10.1371/journal.pone.0044082

**Published:** 2012-09-19

**Authors:** Manuel Varlet, Ludovic Marin, Johann Issartel, R. C. Schmidt, Benoît G. Bardy

**Affiliations:** 1 Movement to Health, EuroMov, Montpellier-1 University, Montpellier, France; 2 Dublin City University, Dublin, Ireland; 3 Department of Psychology, College of the Holy Cross, Worcester, Massachusetts, United States of America; McMaster University, Canada

## Abstract

People often coordinate their movement with visual and auditory environmental rhythms. Previous research showed better performances when coordinating with auditory compared to visual stimuli, and with bimodal compared to unimodal stimuli. However, these results have been demonstrated with discrete rhythms and it is possible that such effects depend on the continuity of the stimulus rhythms (i.e., whether they are discrete or continuous). The aim of the current study was to investigate the influence of the continuity of visual and auditory rhythms on sensorimotor coordination. We examined the dynamics of synchronized oscillations of a wrist pendulum with auditory and visual rhythms at different frequencies, which were either unimodal or bimodal and discrete or continuous. Specifically, the stimuli used were a light flash, a fading light, a short tone and a frequency-modulated tone. The results demonstrate that the continuity of the stimulus rhythms strongly influences visual and auditory motor coordination. Participants' movement led continuous stimuli and followed discrete stimuli. Asymmetries between the half-cycles of the movement in term of duration and nonlinearity of the trajectory occurred with slower discrete rhythms. Furthermore, the results show that the differences of performance between visual and auditory modalities depend on the continuity of the stimulus rhythms as indicated by movements closer to the instructed coordination for the auditory modality when coordinating with discrete stimuli. The results also indicate that visual and auditory rhythms are integrated together in order to better coordinate irrespective of their continuity, as indicated by less variable coordination closer to the instructed pattern. Generally, the findings have important implications for understanding how we coordinate our movements with visual and auditory environmental rhythms in everyday life.

## Introduction

We often move in synchrony with environmental rhythms such as when playing music, applauding or dancing with other people. Understanding the processes underlying the formation and breakdown of stable visual or auditory motor coordination is a key issue today in experimental psychology. Research so far has mainly investigated visual and auditory motor coordination independently of each other, (i) by predominantly using continuous rhythms to investigate visual coordination (e.g., [1]–[5]) but discrete rhythms to investigate auditory coordination (see [6] for a review), and (ii) by manipulating them separately, contrary to most natural cases such as music or dance situations where they are integrated in a unique, multimodal perceptual event. The current study aims to extend the understanding of sensorimotor coordination in more natural situations, which often involve a greater variety of rhythms than the ones investigated in previous research. More specifically, this study examines the dynamics of coordination with an external event contrasting conditions that differ in terms of perceptual modality (visual, auditory), continuity (discrete, continuous), and integration (separated, integrated) factors.

Previous research has shown more efficient sensorimotor coordination with auditory stimuli than with visual stimuli (e.g., [7]–[13]). In these studies, participants were instructed to synchronize the movement of their finger with an auditory metronome or a visual flash while frequency was manipulated. Better performance was obtained with auditory rhythms compared to visual rhythms in unimodal sensory conditions as indicated by more stable coordination for faster stimulus frequencies. The results also revealed better coordination when both visual and auditory rhythms were available (bimodal sensory conditions) corroborating previous research that demonstrated that rhythms from different sensory modalities can be used together to improve motor coordination (e.g., [14]–[16]). However, such an improvement only occurred while visual and auditory rhythms were congruent. Incongruent stimuli affected the stability of the coordination; in that case, greater importance was given to auditory information than to visual information [8], [9]. Overall, these studies show a stronger efficiency and dominance of the auditory modality compared to the visual modality in processes underlying sensorimotor coordination, which have been often explained by its higher temporal resolution (e.g., [17], [18]).

Auditory and visual motor coordination have been compared in all these studies with discrete stimuli; however, contrary to auditory rhythms, visual rhythms in our daily environment are most of the times continuous. Furthermore, although visual motor coordination has been mainly investigated using continuous visual rhythms (e.g., [1]–[5]), the few studies that contrasted continuous and discrete visual rhythms reported better coordination with continuous rhythms, more particularly by using continuous oscillating stimuli [13], [19], [20]. These studies suggest that the continuous and predictable nature of visual rhythms increases coordination, even if this facilitation might be moderated by the spatial component of the rhythms [13]. In contrast and with one exception reporting barely any difference between auditory discrete and continuous rhythms [21], discrete rhythms have been preferred in previous research leaving the influence of its continuity largely unknown. Accordingly, the question can be raised about whether the difference in performance between auditory and visual motor coordination persists with continuous stimuli. Moreover, the continuity of visual and auditory rhythms could also influence their integration. Are visual and auditory continuous rhythms integrated as efficiently as discrete rhythms? Can discrete and continuous information be combined together to improve coordination? For example, while seeing and hearing another individual applauding, does the availability of both discrete sound of claps and continuous visual information of hands' movements improve our ability to coordinate with them compared to respective unimodal sensory conditions? The goal of the current study was to address such questions. More specifically, this study aimed at investigating whether sensorimotor coordination with unimodal visual and auditory rhythms, or with bimodal visuo-auditory rhythms depend on their continuity.

In addition to the missing comparisons of important conditions, it is difficult to contrast results across existing studies in the literature due to several differences in experimental conditions. The first issue creating differences is the variety of the tasks used (e.g., tapping or oscillation) that creates differences in the continuity of participants' movement and influences the control processes underlying the coordination [22]–[26]. Investigations of between cycles dynamics have revealed an event-based form of timing in synchronization of discontinuous movements and an emergent form of timing in synchronization of continuous movements, characterized by a negative or non-negative lag 1 autocorrelation (respectively) of the series of movement's periods [22], [23]. In accordance with the Wing and Kristofferson (1973) model, it has been assumed that the event-based form of timing implies an explicit representation of the temporal goal by an internal timekeeper, which allows determining cognitive events that trigger motor responses [22]–[28]. The execution of each response is proposed to be affected by a random (white noise) motor delay, resulting in differenced white noise in the successive periods, and thus, a negative lag 1 autocorrelation. Inversely, no explicit representation of time is needed in the emergent form of timing. Timing is assumed to come from a continuous modulation of the dynamical properties of the effector's movement, the successive periods to be affected by simple white noise, resulting in a non-negative lag 1 autocorrelation.

Furthermore, examinations of within cycles dynamics of tasks that differ in the continuity of participants' movements have also shown that the limit cycle (position vs. velocity) of synchronized discontinuous movements has an important nonlinearity and asymmetry whereas the limit cycle of continuous movements is nearly circular with a slight asymmetry at the point where the pacing signal occurs [22]. These studies have shown the necessity to compare results involving the same kind of movement and to examine the between and within cycles dynamics of participants' movement to further understand sensorimotor coordination. Accordingly, it is possible that these two dynamics depend not only on the movement continuity of participants but also on the continuity and perceptual modality of the stimuli.

The second issue creating differences when contrasting results from previous studies is the use of different stimulus frequencies. The frequency of the rhythm has an important influence on the coordination produced by participants. For example, the stability of the coordination, how the movement of participants precedes or lags the stimulus as well as its continuity change as a function of the stimulus frequency [5], [29]–[31]. Because movement frequency influences sensorimotor coordination, it is necessary to contrast results obtained for the same frequency and also to examine the influence of the other investigated factors on different frequencies. It is possible for instance that both continuity and perceptual modality of the stimuli influence sensorimotor coordination only for specific frequencies.

Accordingly, specific experimental conditions and analyses were used in the current study to investigate the influence of the continuity of visual and auditory rhythms on unimodal and bimodal sensorimotor coordination. We examined the coordination dynamics of synchronized continuous oscillatory movements with visual and auditory stimulus rhythms at different frequencies, which could be either unimodal (visual or auditory) or bimodal (both visual and auditory), as well as discrete or continuous. We expected an influence of the continuity of visual and auditory rhythms on the coordination produced by participants. In unimodal sensory conditions, better auditory motor coordination was expected to occur only with discrete rhythms. We also expected better coordination in bimodal sensory conditions compared to unimodal sensory conditions in line with previous research. However, it is possible that such an improvement depends on the continuity of the stimulus rhythms.

## Methods

### 1. Ethics statement

All participants provided written informed consent prior to the experiment, which was approved by the local Ethics Committee (CPP Sud Méditérannée III, Montpellier, France, AFSSAPS 2009-A00513-54 24, 07/22/2009) and conformed to the Declaration of Helsinki.

### 2. Participants

Fifteen adults (eight females and seven males) volunteered to participate in the experiment. Their mean age was 26.7 (*SD* = 5.8) and all reported normal or corrected-to-normal vision as well as no history of auditory and movement disorders. Thirteen of our participants had no musical training. The two who did no longer actively played music.

### 3. Materials

**Figure 1:**
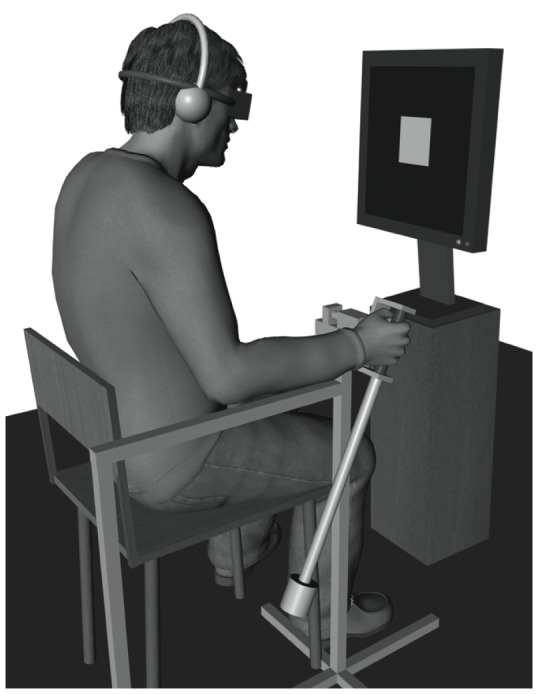
Experimental setup.

Participants sat in a chair, wore headphones and wore soldering glasses that prevented them seeing their movements while ensuring that they could see the target displayed in front of them on a 19″ LCD monitor with a frame refresh rate of 75 Hz (Lenovo, Morrisville, NC, USA) (Figure 1). They swung a wrist pendulum with a length of 60 cm and a mass of 150 g attached at the bottom, which was fixed on a metallic structure that allowed only front to back oscillatory movements. The pendulum's eigenfrequency was 0.75 Hz. A potentiometer measured the angular displacements of the pendulum during the trials at a sampling rate of 50 Hz.

A computer recorded the movements of participants and controlled the stimuli. The visual stimulus consisted of a square (10 cm×10 cm) changing from black to red, displayed at eye level on a black background. For the discrete stimuli, the square flashed from black to red during 80 ms and for the continuous stimuli, the square faded continuously and sinusoidally from black to red. The discrete auditory stimuli consisted of a 80 ms tone at a frequency of 800 Hz and the continuous stimuli consisted of a continuous tone, which sinusoidally modulated from 400 Hz to 800 Hz.

### 4. Task

Participants were instructed to oscillate the pendulum and to synchronize the adduction of their wrist with the stimuli. For discrete stimuli, they had to synchronize the maximal adduction of their wrist with the flashes or the tones; and for continuous stimuli, they had to synchronize the maximal adduction of their wrist when the red was the most salient or when the sound was the most acute (i.e., 800 Hz). They had to maintain the coordination between the pendulum and the stimulus in eight different stimulus combinations: VD (Visual Discrete), VC (Visual Continuous), AD (Auditory Discrete), AC (Auditory Continuous), VD-AD (Visual and Auditory Discrete), VC-AC (Visual and Auditory Continuous), VD-AC (Visual Discrete and Auditory Continuous), and VC-AD (Visual Continuous and Auditory Discrete). The combined stimuli were always congruent, which means that they were in phase with one another. In other words, the points in time at which the participants were instructed to synchronize with of the two combined rhythms were identical. We used three different frequencies for the stimuli (0.5, 0.75 and 1.0 Hz) that extended across the average preferred movement frequency range obtained in a pilot experiment with ten participants. Participants also performed an additional trial in a self-paced condition in which they were instructed to perform oscillations at their most comfortable tempo, with no external stimulus. This trial was added in order to examine the participants' spontaneous movement dynamics.

### 5. Procedure

Participants were informed that the experiment was being conducted in order to investigate visual and auditory motor coordination and that they were required to synchronize the swinging of the pendulum with visual and auditory stimuli, which could be discrete or continuous. They were told that the stimuli would be congruent in all experimental conditions. They were given a practice period to explore the synchronization with the different stimulus combinations and frequencies. Then, participants were engaged in the experimental session and performed twenty-four randomized trials (3 frequencies by 8 stimulus combinations) of 60 s with a 5-minute break half way through the trials. They were instructed to do their best to keep their movement synchronized with the stimuli in all the trials. At the end of the session, they performed the self-paced control trial of 60 s without any stimulus.

### 6. Design and analysis

We discarded the first 5 s of each trial to eliminate transient behavior. Angular displacements of participants' oscillations were centered around zero and low-pass filtered using a bi-directional 10 Hz Butterworth filter. The minima and maxima of the time series were extracted. We normalized the time series between −1 and 1 and computed different measures relating to synchronization performance as well as between and within cycles dynamics.

#### Synchronization performance

To evaluate synchronization, we computed the discrete relative phase between the stimulus and maximal adductions of participants to examine their performance [32]. The discrete relative phase was computed at each cycle between 0° and 360° by taking as references the maximal movement adduction of participants and (i) the onset of the discrete stimulus (i.e., VD and AD) and (ii) the maximal peak of the modulating sinusoidal signal of the continuous stimulus (corresponding to the synchronization point, i.e., the most salient red for VC and the most acute sound for AC), which were the same when combined because of their congruency.

We used circular statistics to compute the average and standard deviation of the relative phase [33]. Using the target as reference, positive values of average relative phase indicated movements following the stimulus and negative values indicated movements preceding the stimulus. The standard deviation of relative phase was used to investigate the coordination variability produced by the participants. Perfect coordination is indicated by average relative phase and standard deviation values equal to zero.

#### Between cycles dynamics

As suggested by previous research that focused on between cycles dynamics, event-based and emergent forms of timing can be distinguished on the basis of the negative or non-negative lag 1 autocorrelation of the series of movement periods [22], [23]. To investigate the forms of timing involved in our different experimental conditions, we extracted movement periods as the time between maximal adduction moments and then computed the autocorrelation functions of the series.

#### Within cycles dynamics

In view of the cycle asymmetry reported in the literature, our within cycles dynamics analysis focused separately on the half-cycles *To* and *Away* of the stimulus [22], [34]. The half-cycle *To* was defined as the movement going from maximal abduction to maximal adduction (reversal point to synchronize); and the half-cycle *Away* was defined as the movement going from maximal adduction to maximal abduction. We computed first the duration of the half-cycles *To* and *Away*, which was expressed as a percentage of the movement period, in order to compare the different frequency conditions. A percentage of 50% for the half-cycles *To* and *Away* indicates perfect symmetry between the two movement half-periods. We also computed the average normalized limit cycle across participants in the different conditions to investigate the movement trajectory (e.g., [22], [35]). The velocity of the wrist pendulum oscillation was computed from its angular position and normalized between −1 and 1 by dividing it by 2π/*p*, where *p* is the movement period. Position and velocity of each half-cycle *To* and *Away* were then time normalized to 30 points using a spline interpolation procedure and all half-cycles of the trial were then averaged point by point. To complete the graphical examination of the limit cycles, we calculated the deviation from a straight line in the average normalized Hooke's portraits representing the contribution of nonlinear (*NL*) components, which can be quantified by *NL* = 1−*r* ^2^ where *r* ^2^ is the amount of variance explained by the linear regression of position onto acceleration and attributed to a harmonic oscillation (e.g., [29], [35], [36]). Higher values of *NL* indicate higher nonlinearity in movement trajectory.

### 7. Statistical analysis

To compare the coordination of participants in unimodal (i.e., VD, VC, AD and AC) and bimodal conditions (i.e., VD-AD, VC-AC, VD-AC and VC-AD), we used repeated-measures ANOVAs with Stimulus Frequency (0.5, 0.75 and 1.0 Hz) and Mode (Unimodal, Bimodal) as factors. Then we performed two additional statistical analyses. First, to investigate the influence of the rhythm continuity and sensory modality, unimodal conditions were submitted to repeated-measures ANOVAs with Stimulus Frequency (0.5, 0.75 and 1.0 Hz), Sensory Modality (Visual and Auditory) and Stimulus Continuity (Discrete and Continuous) as factors. Second, bimodal conditions were submitted to repeated-measures ANOVAs with Stimulus Frequency (0.5, 0.75 and 1.0 Hz) and Stimulus Combination (VD-AD, VC-AC, VD-AC and VC-AD) as factors in order to examine whether the integration of visual and auditory rhythms depended on their continuity. These analyses were performed on the average and standard deviation of relative phase variables, and the factor Half-cycle (*To* and *Away*) was added for the ANOVAs on the duration and trajectory nonlinearity (*NL*) of the movement half-cycles. Bonferroni post-hoc tests were used when necessary to investigate the details of significant effects. We also used one-sample *t*-tests to determine the differences from zero of the average relative phase and of the lag 1 autocorrelation, and paired *t*-tests for the within cycles dynamics analysis of self-paced oscillations.

## Results

### 1. Self-paced oscillations

#### Between cycles dynamics

**Figure 2:**
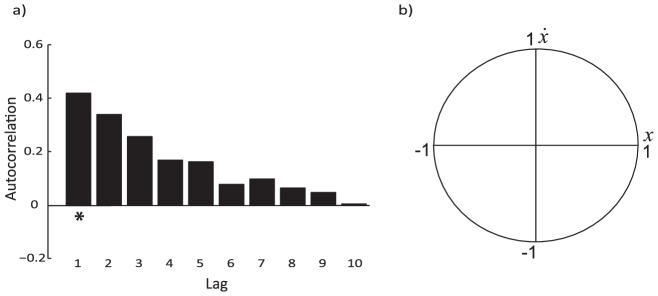
Within and between cycles dynamics in the self-paced condition. Mean autocorrelation function of periods (a) and mean limit cycle (b) produced by participants. The *Asterisk* indicates a significant difference from zero for the lag 1 autocorrelation.

The analysis of the participants' movement period in the self-paced condition revealed an average preferred frequency of 0.81 Hz (*SD* = 0.13) and a significant positive lag 1 autocorrelation (*p*<.05) indicating the involvement of an emergent form of timing as expected for a continuous oscillatory movement (Figure 2a).

#### Within cycles dynamics

Paired *t*-tests revealed no differences in the duration (*t*(14) = 0.32, *p*>.1) and *NL* (*t*(14) = 0.72, *p*>.1) between the half-cycles of participants' movement and the average limit cycle showed a shape close to a circle (Figure 2b), indicating important continuity and no asymmetry in the movement when participants oscillated the pendulum at their preferred tempo.

### 2. Synchronized oscillations

#### Synchronization Performance

**Figure 3:**
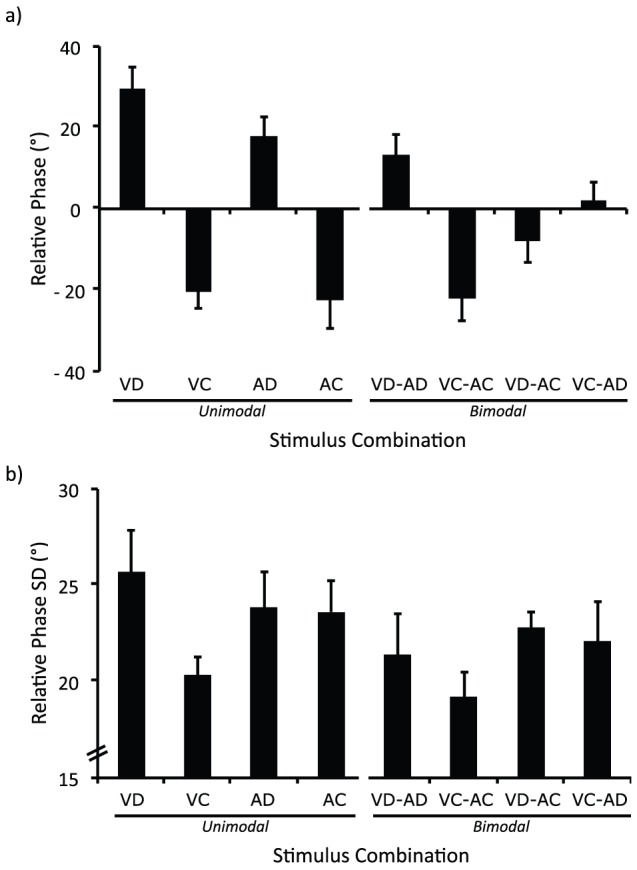
Performance for synchronized oscillations. Relative phase (a) and its standard deviation (b) as a function of stimuli combination. *Error bars* represent standard errors.

The 3 (Stimulus Frequency)×2 (Mode) ANOVA on average relative phase yielded a significant main effect for Stimulus Frequency (*F*(2, 118) = 15.39, *p*<.05, η^2^ = 0.21) indicating phase shifts values from the intended coordination increasing with faster frequencies (all frequencies significantly different (*p*<.05)). One-sample *t*-tests indicated phase shift values that were not significantly different from zero when stimulus frequency was close to the preferred frequency (i.e., 0.75 Hz) of participants (*M* = −2.77; *SD* = 32.57) (*t*(119) = −0.93, *p*>.1), and phase shift values that were significantly lower (*M* = −8.78; *SD* = 30.26) and higher (*M* = 7.29; *SD* = 34.58) than zero for stimulus frequencies 0.5 Hz (*t*(119) = −3.18, *p*<.05) and 1.0 Hz (*t*(119) = 2.31, *p*<.05), respectively. In line with previous research, these results indicate that participants preceded and followed the stimulus while its frequency was respectively slower and faster than their preferred frequency (e.g., [1]). This ANOVA did not reveal any other significant effects (all *p values*>.1). To further examine whether better coordination occurred in bimodal compared to unimodal conditions, we performed a 3 (Stimulus Frequency)×2 (Mode) ANOVA on the *absolute* values of the average relative phase. This analysis yielded a significant main effect for Mode (*F*(1, 59) = 7.12, *p*<.05, η^2^ = 0.11) showing that participants were closer to the intended coordination (i.e., closer to a zero phase shift) when both visual and auditory rhythms were available (Figure 3a). This ANOVA did not reveal any other significant effects (all *p values*>.1).

For unimodal conditions, the 3 (Stimulus Frequency)×2 (Sensory Modality)×2 (Stimulus continuity) ANOVA on average relative phase revealed a significant main effect for Stimulus Frequency (*F*(2, 28) = 7.90, *p*<.05, η^2^ = 0.36) in line with the previous analysis, and a significant main effect for Stimulus Continuity (*F*(1, 14) = 59.99, *p*<.05, η^2^ = 0.81) yielding higher phase shift values for discrete compared to continuous stimuli. One-sample *t*-tests indicated phase shift values significantly higher (*t*(89) = 7.63, *p*<.05) or lower (*t*(89) = −7.04, *p*<.05) than zero for discrete and continuous stimuli, respectively. Interestingly, the analysis also showed a significant interaction between Sensory Modality and Stimulus Continuity (*F*(1, 14) = 4.78, *p*<.05, η^2^ = 0.25) revealing a phase shift closer to the intended coordination with the auditory compared to visual modality but only for discrete rhythms (*p*<.05). This ANOVA and the one performed on *absolute* values of the average relative phase did not reveal any other effects (all *p values*>.1).

For bimodal conditions, the 3 (Stimulus Frequency)×4 (Stimulus Combination) ANOVA on average relative phase also showed a significant main effect for Stimulus Frequency (*F*(2, 28) = 6.11, *p*<.05, η^2^ = 0.30) supporting the previous analyses, and a significant main effect for Stimulus Combination (*F*(3, 42) = 12.00, *p*<.05, η^2^ = 0.46). One-sample *t*-tests indicated phase shift values significantly higher than zero with discrete rhythms (i.e., VD-AD) (*t*(44) = 3.39, *p*<.05), lower than zero with continuous rhythms (i.e., VC-AC) (*t*(44) = −6.18, *p*<.05), and not different from zero when discrete and continuous rhythms were combined (i.e., VD-AC and VC-AD) (*t*(44) = −1.98, *p*>.1 and *t*(44) = 0.43, *p*>.1, respectively). These results demonstrate that participants followed discrete stimuli but led continuous ones, a behavior that was found to disappear when discrete and continuous stimuli were combined. This ANOVA and the one performed on *absolute* values of the average relative phase did not reveal any other effects (all *p values*>.1).

The 3 (Stimulus Frequency)×2 (Mode) ANOVA on the standard deviation of relative phase yielded a significant main effect for Mode (*F*(1, 59) = 7.12, *p*<.05, η^2^ = 0.11) indicating lower variability in bimodal conditions compared to unimodal conditions (Figure 3b). For unimodal conditions, the 3 (Stimulus Frequency)×2 (Sensory Modality)×2 (Stimulus Continuity) ANOVA showed a significant interaction between Sensory Modality and Stimulus Continuity (*F*(1, 14) = 8.24, *p*<.05, η^2^ = 0.37) indicating lower variability with continuous visual rhythm (VC) compared to discrete visual rhythm (VD) (*p*<.05). For bimodal conditions, the 3 (Stimulus Frequency)×4 (Stimulus Combination) ANOVA did not show any significant effect. These results show that the continuity of the rhythms influenced the stability of visual but not auditory motor coordination and that more stable coordination occurred when both visual and auditory rhythms were available, irrespective of their continuity. These ANOVAs did not reveal any other effects (all *p values*>.1).

Together, these results demonstrate that the continuity of the rhythms influenced the performance of both visual and auditory motor coordination, but with a stronger effect observed for visual stimuli. As expected, these results also demonstrate better performances with auditory compared to visual modality only for discrete rhythms and with bimodal compared to unimodal stimuli. The facilitation in bimodal conditions was irrespective of the continuity of the combined rhythms, even if it is important to note that combining discrete and continuous rhythms together (i.e., VD-AC and VC-AD) allowed participants to be closer to the instructed coordination (i.e., phase shifts not different from zero).

#### Between cycles dynamics

**Figure 4:**
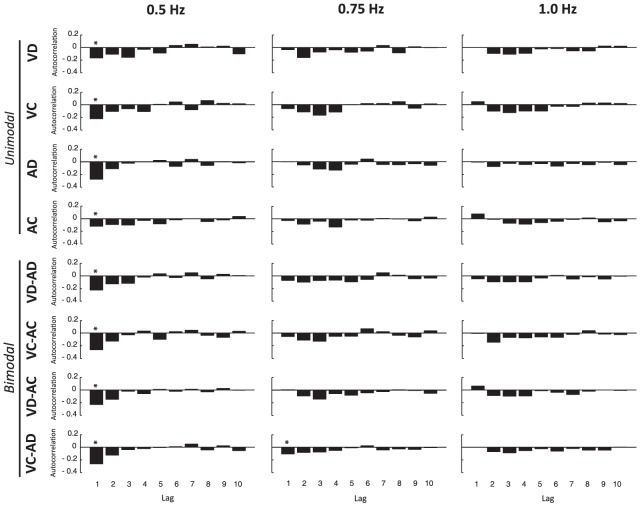
Mean autocorrelation functions for synchronized oscillations as a function of stimulus frequency and stimulus combination. *Asterisks* indicate significant differences from zero for the lag 1 autocorrelation.

One-sample *t*-tests showed no differences from zero for the lag 1 autocorrelation for all stimuli combinations at 1 Hz (*p*>.1) and at 0.75 Hz (*p*>.1), except for VC-AD showing a negative lag 1 (*p*<.05). This result supports previous findings having demonstrated the involvement of an emergent form of timing in synchronized oscillations [22], [23] (Figure 4). However, significant negative lag 1 autocorrelation (*p*<.05) were found for all combinations of stimuli at 0.5 Hz, indicating that participants switched to an event-based form of timing for slower stimulus frequencies. The unequal number of cycles across conditions due to the manipulation of the stimulus frequency may have influenced these results, and for this reason we performed the same analysis while using only the last twenty-five cycles of each trial. The results (not reported here) were identical statistically.

The analysis of the autocorrelation functions showed an influence of the stimulus frequency on the form of timing involved during visual and auditory motor coordination. However, no significant influences of the continuity and perceptual modality of the stimuli were found.

#### Within cycles dynamics

**Figure 5:**
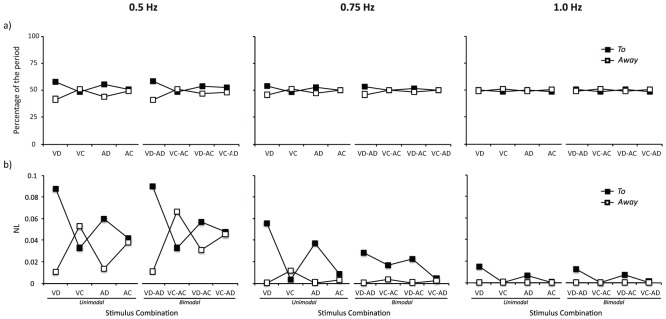
Within cycles dynamics for synchronized oscillations. Half-cycle duration expressed as a percentage of movement period (a) and non-linearity (NL) in movement trajectory (b) of the half-cycles *To* (black) and *Away* (white) for the different stimulus combinations in slow (left), intermediate (middle) and fast (right) stimulus frequency.

The 3 (Stimulus Frequency)×2 (Mode)×2 (Half-cycle) ANOVA on half-cycles duration yielded a significant interaction between Stimulus Frequency and Half-cycle (*F*(2, 118) = 10.78, *p*<.05, η^2^ = 0.15) (Figure 5a). Post-hoc analyses revealed longer duration for the half-cycle *To* compared to the half-cycle *Away* at 0.5 Hz (*p*<.05). For the sake of clarity, we performed separate ANOVAs for each movement frequency in subsequent analyses. For unimodal conditions, a 2 (Sensory Modality)×2 (Stimulus Continuity)×2 (Half-cycle) ANOVA showed a significant interaction between Half-cycle and Stimulus Continuity for 0.5 Hz (*F*(1, 14) = 7.00, *p*<.05, η^2^ = 0.33) and for 0.75 Hz (*F*(1, 14) = 7.01, *p*<.05, η^2^ = 0.34), indicating longer half-periods *To* for these frequencies when coordinating to discrete stimuli (i.e., VD and AD). For bimodal conditions, a 4 (Stimulus Combination)×2 (Half-cycle) ANOVA revealed a significant interaction between these two factors for 0.5 Hz (*F*(3, 42) = 6.91, *p*<.05, η^2^ = 0.33) and for 0.75 Hz (*F*(3, 42) = 6.40, *p*<.05, η^2^ = 0.31). Post-hoc comparisons yielded longer half-cycle *To* for the conditions VD-AD and VD-AC at 0.5 Hz (*p*<.05) and for the condition VD-AD at 0.75 Hz (*p*<.05). These results confirm longer half-cycles *To* with discrete slow rhythms and show that this effect was stronger for visual rhythms as indicated by asymmetries occurring even when combined with continuous auditory rhythms (i.e., VD-AC). These ANOVAs did not reveal any other effects (all *p values*>.1).

The 3 (Stimulus Frequency)×2 (Mode)×2 (Half-cycle) ANOVA on half-cycles nonlinearity (*NL*) yielded a significant main effect for Half-cycle (*F*(2, 118) = 10.78, *p*<.05, η^2^ = 0.15), indicating greater *NL* for the half-cycle *To* compared to the half-cycle *Away*, and a significant main effect for Stimulus Frequency (*F*(2, 118) = 10.78, *p*<.05, η^2^ = 0.15), indicating greater *NL* for 0.5 Hz compared to 0.75 Hz and 1.0 Hz (*p*<.05). For unimodal conditions, a 3 (Stimulus Frequency)×2 (Sensory Modality)×2 (Stimulus Continuity) ANOVA performed on the different movement frequencies showed a significant interaction between Half-cycle and Stimulus Continuity for 0.5 Hz (*F*(1, 14) = 4.59, *p*<.05, η^2^ = 0.26) and for 0.75 Hz (*F*(1, 14) = 6.05, *p*<.05, η^2^ = 0.30), indicating greater *NL* of the half-cycle *To* when coordinating to discrete rhythms (i.e., VD and AD). For bimodal conditions, the 4 (Stimulus Combination)×2 (Half-cycle) ANOVA revealed a significant interaction between these two factors for 0.5 Hz (*F*(3, 42) = 5.07, *p*<.05, η^2^ = 0.27) as well as for 0.75 Hz (*F*(3, 42) = 3.11, *p*<.05, η^2^ = 0.18). Post-hoc comparisons yielded greater *NL* for the half-cycle *To* in the condition VD-AD at 0.5 Hz (*p*<.05) and in the conditions VD-AD and VD-AC at 0.75 Hz (*p*<.05). These results show that greater movement nonlinearity accompanied the longer duration of the half-cycle *To* with discrete rhythms, which was also stronger for visual rhythms as indicated by *NL* asymmetries persisting even when combined with continuous auditory rhythms (i.e., VD-AC). These ANOVAs did not reveal any other effects (all *p values*>.1).

**Figure 6:**
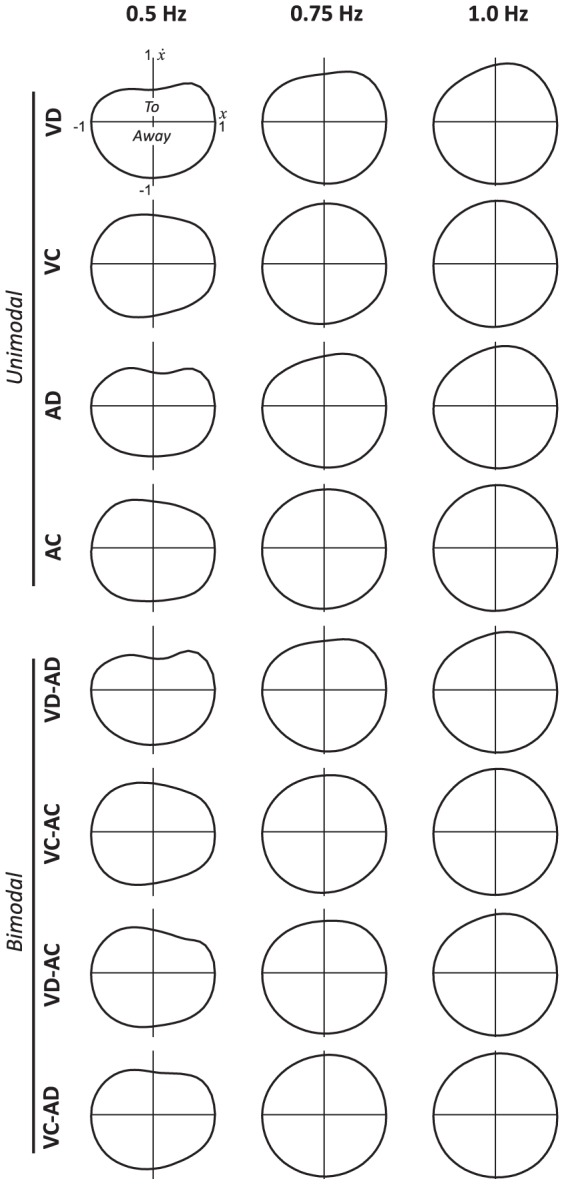
Averaged normalized limit cycles for the different stimulus combinations as a function of stimulus frequency, with *x* and 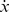 representing the position and velocity of wrist pendulum oscillations of participants, respectively.

The graphical analysis of the averaged limit cycles confirmed the previous results (Figure 6). The continuity of participants' movement increased with faster frequencies, as revealed by nearly circular limit cycles, and a stronger nonlinearity was observed for the half-cycle *To* at 0.5 Hz in conditions with discrete rhythms (i.e., VD, AD and VD-AD). Further explorations showed that this nonlinearity with discrete stimuli was characterized by an asymmetry at the stimulus occurrence point. Although this effect decreased with faster frequencies, a slight asymmetry was still observed, in line with previous results reported for synchronized oscillations [22]. Generally, such effects were not observed while continuous rhythms were available, even if asymmetries remain observable when combined with discrete visual rhythms (i.e., see the condition VD-AC at 1.0 Hz).

## Discussion

The aim of the current study was to better understand the influence of rhythm continuity (i.e., whether it is discrete or continuous) on visual and auditory motor coordination. In particular, we investigated whether the better performance previously observed for coordinating with auditory compared to visual stimuli and for coordinating with bimodal compared to unimodal stimuli depends on the continuity of the stimulus rhythms. We examined the synchronized oscillations of a wrist pendulum with auditory and visual stimulus rhythms which had different frequencies, and were either discrete or continuous and unimodal or bimodal. As expected, the results demonstrate differences when coordinating with discrete and continuous rhythms, and moreover, that the better performance observed with auditory and bimodal stimuli depend on the continuity of the stimulus rhythms. We now discuss these results in turn.

### 1. Discrete vs. continuous rhythms

Previous research investigated visual and auditory motor coordination irrespective of the continuity of the rhythms [1]–[6]. Across these studies, similar properties have been revealed for the coordination with discrete and continuous rhythms that might lead one to think that the same processes are involved. However, the results of the current study show that the continuity of the stimulus rhythm strongly influences both visual and auditory motor coordination.

Independently of the perceptual modality, the results demonstrate that the continuity of the stimulus rhythm influences the phase shift from the intended coordination. The movements of participants preceded continuous stimuli but followed discrete stimuli. This result, confirming an effect that was previously suggested in a study that compared continuous and intermittent rhythms in visual motor coordination [20], represents an important finding. Because the preferred movement frequency of participants (i.e., 0.81 Hz) was faster than our middle stimulus frequency (i.e., 0. 75 Hz), the movement leading the continuous stimuli corroborates previous research that investigated the synchronized oscillations of a wrist pendulum to continuous visual rhythms [1]. The movement lagging the discrete stimuli is also in line with studies that investigated auditory motor coordination with a slow metronome and may indicate that participants used reactive responses [37].

The results also demonstrate that the continuity of visual and auditory stimuli influence the within cycles dynamics of participants' movements. A longer duration and a stronger nonlinearity (toward the location of stimulus occurrence) were observed for the half-cycle *To* compared to the half-cycle *Away* while synchronizing with slow discrete rhythms. According to previous research that showed a better timing for shorter movement duration toward the target [34], these within cycles differences, and more specifically the longer half-cycle *To* found with discrete visual rhythms, might explain the greater coordination variability observed in the condition VD compared to VC.

However, although it has been demonstrated that between and within cycles dynamics are not independent from each other and that the specific movement trajectories contribute to the achievement of the different forms of timing [22], [34], differences in movement continuity of participants observed between discrete and continuous stimuli did not result in differences in their between cycles dynamics. It is however important to note that the autocorrelation functions analysis showed the involvement of an emergent form of timing for 0.75 and 1 Hz in accordance with previous studies that investigated synchronized oscillations [22], [23], but that participants switched to an event-based form of timing at 0.5 Hz for both discrete and continuous stimuli. This new finding not only supports the view that movement frequency influences timing [30] but also connects frequency-induced changes in movement continuity of participants to changes in their between cycles dynamics. It is possible that the stronger movement discontinuity (*NL*) observed at 0.5 Hz made more difficult the continuous modulation of the dynamical properties of the pendulum to coordinate and led participants to adopt an event-based form of timing [22]–[26].

### 2. Visual vs. auditory rhythms

Often investigated independently of each other, little is known about visual vs. auditory modalities for sensorimotor coordination. The results of the current study extend our understanding by showing that the influence of the continuity of the stimulus rhythm is stronger for visual than for auditory motor coordination. Although we found for both an effect of the rhythm continuity on the phase shift and within cycles dynamics of participants (as discussed above), visual motor coordination was more affected as indicated by greater variability with discrete as compared to continuous rhythms (not observed for auditory motor coordination). These results support previous studies that found better coordination with continuous oscillating visual rhythms compared to light flashes [13], [19], [20], and show that this facilitation can also occur for rhythms without spatial component such as fading stimuli. An explanation for such results may be that, contrary to auditory rhythms that may be either discrete (e.g., steps or claps produced by someone) or continuous (e.g., sliding sound of arm movements on a table) in our daily environment, visual rhythms are most of the time continuous, and thus, participants may be better at coordinating with continuous visual rhythms.

Therefore, this result is in accordance with our expectation that the better performance reported for auditory compared to visual motor coordination may be due to the use of discrete rhythms in these studies [7]–[12]. When comparing visual and auditory motor coordination with discrete rhythms, our results corroborate the findings of these studies as indicated by a larger phase shift from the intended coordination, together with stronger asymmetries of the visually synchronized oscillations. More interesting, our results demonstrate that the differences between visual and auditory sensory modalities vanished with continuous rhythms. Accordingly, our results suggest that the lower temporal resolution of the visual modality affects the control processes only when coordinating with discrete rhythms (e.g, [17], [18]).

### 3. Unimodal vs. bimodal rhythms

Although in most natural cases visual and auditory rhythms are integrated in a unique multimodal perceptual event, only a small number of studies have investigated motor coordination with both visual and auditory rhythms available [7]–[12]. Except for these studies that showed a dominance of auditory over visual information and that discrete visual and auditory rhythms become integrated when congruent, the multimodal processes underlying motor coordination remain largely unknown. In this study, we examined whether visual and auditory rhythms can be also integrated when they are both continuous and when they have different continuity (i.e., both discrete and continuous).

In line with previous research, the results demonstrate better coordination in bimodal sensory conditions compared to unimodal sensory conditions [11], [14]–[16]. The coordination performed by participants in bimodal conditions was found to be more stable and closer to the instructed coordination. Our results demonstrate better performance in bimodal discrete condition corroborating previous findings [11], but also, for the first time, when auditory and visual rhythms were both continuous and when they had different continuity. Interestingly, when visual and auditory rhythms had different continuity, the results also show that participants were better able to maintain the instructed coordination. Whereas the movement of participants led continuous stimuli and followed discrete stimuli, we found shifts closer to zero when discrete and continuous stimuli were combined. Generally, these results reveal that continuous rhythms and rhythms of different continuity can be integrated to the same level as discrete rhythms in order to improve sensorimotor coordination.

Moreover, because discrete and continuous rhythms appear to equally influence the coordination in bimodal conditions independently of their sensory modality, the stronger influence of the auditory modality reported in previous studies seems not to be observed (e.g., [8], [9]). This may be due to the coordination task used in the current study. In fact, the continuous nature of the wrist pendulum oscillations might have supported the use of visual rhythms that are usually continuous in our environment, attenuating the often-observed dominance of the auditory modality. These results might have been different if we had used a discontinuous movement such as a tapping movement.

Other factors may also be involved, such as the saliency of our stimuli. Using less salient stimuli (e.g., smaller visual and quieter auditory stimuli) may have revealed more differences within and between unimodal and bimodal conditions [38]. The difference may also have been stronger if we had used more difficult tasks such as coordinating at faster frequencies. Moreover, it is possible that the effects could have been stronger if we had manipulated the congruency of the rhythms. It has been demonstrated that the information transmission is slower for the visual modality than for the auditory modality, and consequently, manipulating the delay of the stimuli could improve their congruency, and thus, the coordination in bimodal conditions (e.g., [39], [40]). Finally, using rhythms having spatial information may have changed the integration of visual and auditory stimuli as well. In fact, visual modality has a better spatial resolution and could have a stronger influence when the coordination requires the use of spatial information (e.g., [41], [42]). We consider these different questions as important future research directions.

Using comparable experimental conditions and analyses, the current study shows clear differences between visual and auditory, between discrete and continuous, and between unimodal and bimodal external rhythms as well as different interactions between these factors. Our results have implications for future research aiming to understand the etiology of sensorimotor coordination with environmental rhythms. They support existing research showing a variety of control processes allowing the coordination of our movements with environmental rhythms [22]–[26]. However, contrasting with these studies that mainly focused on the influence of the kind of movement performed, our results show the influences of the *properties* of the environmental rhythms. They corroborate and further detail the influences of the frequency and sensory modality of the rhythms shown before [1]–[11], but more interestingly, thanks to the continuity effect demonstrated, extend the growing recent literature showing influences of other rhythms' properties such as their variability, their spatial compatibility, their amplitude and even their social nature [13], [43]–[46]. We observed a facilitation effect of continuous rhythms for visual motor coordination with an external event, a result that encourages the use of continuity of a visual stimulus when coordination needs to be efficient. These results also have implications for future research aiming to model the underlying processes of sensorimotor coordination. For example, different modeling attempts have accounted for sensorimotor coordination by using a continuous coupling between participants' movement and the stimulus, irrespective of its discrete or continuous nature (e.g., [47], [48]). The differences between discrete and continuous rhythms observed in the current study may have to be taken into account in future models intending to understand how people coordinate their movements with external events in everyday life.
